# Anti-mullerian hormone (AMH) in pregnant buffaloes: maternal and fetal plasma concentrations and immunolocalization in the fetal gonads

**DOI:** 10.1590/1984-3143-AR2023-0152

**Published:** 2025-02-24

**Authors:** Nathalia Nogueira da Costa, Maria Auxiliadora Pantoja Ferreira, Priscilla do Carmo Azevedo Ramos, Alessandra Ximenes Santos, Marcela da Silva Cordeiro, Priscila di Paula Bessa Santana, Vanessa Cunha Brito, Thiago Velasco Guimarães Silva, Bruno Porpino Homobono, Eduardo Baia de Souza, William Allan King, Moyses Santos Miranda, Simone Socorro Damasceno Santos

**Affiliations:** 1 Laboratório de Fertilização in vitro Prof. Dr. Otavio Mitio Ohashi, Instituto de Ciências Biológicas, Universidade Federal do Pará, Belém, PA, Brasil; 2 Laboratório de Imunohistoquimica e Biologia do Desenvolvimento, Universidade Federal do Pará, Belém, PA, Brasil; 3 Instituto Federal do Pará, Ananindeua, PA, Brasil; 4 Instituto Socioambiental e de Recursos Hidricos, Universidade Federal Rural da Amazônia, Belém, PA, Brasil; 5 Instituto de Medicina Veterinária, Universidade Federal do Pará, Castanhal, PA, Brasil; 6 Department of Biomedical Sciences, Ontario Veterinary College, University of Guelph, Guelph, Ontario, Canada

**Keywords:** AMH, buffalo cows, buffalo fetus

## Abstract

Anti-Müllerian Hormone (AMH), a member of the TGF-β superfamily, plays a critical role in mammalian fertility. This study aimed to investigate AMH concentrations in pregnant buffalo cows and their fetuses, and to characterize the cellular distribution and immunolocalization of AMH within fetal gonads. Gonads were collected from 12 male and 14 female buffalo fetuses, fixed in 10% buffered formalin, and processed for routine histological analysis and immunohistochemical localization of AMH. Blood samples were collected from 26 pregnant buffalo cows and their corresponding fetuses into EDTA-coated tubes. AMH levels in both maternal and fetal blood were quantified using a commercially available bovine AMH ELISA kit. Significant (p < 0.05) inter-individual variations in maternal and fetal AMH concentrations were observed. In males, fetal AMH concentrations increased significantly with gestational age, with levels substantially higher compared to females at all gestational ages (52-1222 times higher). Immunohistochemistry revealed intense AMH immunoreactivity in the cytoplasm of pre-Sertoli cells in male fetuses. In females, no significant correlation was found between fetal AMH concentrations and follicular populations, although intense AMH immunostaining was observed in the cytoplasm of oocytes within primordial follicles. These findings suggest that fetal AMH concentrations in buffaloes are influenced by gestational age and sex, and that fetal AMH likely does not readily cross the placental barrier. Further investigations are warranted to elucidate the intricate role of AMH in gonadal development and function in buffaloes.

## Introduction

Discovered by Alfred Jost in 1940, the anti-Müllerian hormone (AMH) is a140-kDa glycoprotein encoded by a 275-bp long gene located on the short arm of chromosome 19 in humans (Jost, 1946; Cate et al., 1986; Imbeaud et al., 1994; Picard et al., 1986). AMH belongs to the TGF-β superfamily, which also includes TGF-β, inhibin, activin and GDF-9, all involved in gonadal development and fertility regulation (Josso and di Clemente, 1999). AMH plays a crucial role in the regression of female tubular genitalia in the male fetus (Jost et al., 1973). In humans, AMH production by Sertoli cells from the sixth week of gestation leads to irreversible regression of the Müllerian ducts, a process completed by the end of the ninth week (Rajpert-De Meyts et al., 1999; Rey et al., 2003). Except for a transient decline in the perinatal period, AMH secretion remains high until puberty, when Sertoli cell maturation is characterized by a decrease in AMH levels, as observed in a human study (Josso et al., 1993).

In addition to its well-known function in male development, AMH exerts other inhibitory effects on the reproductive tissues of both sexes (Josso and di Clemente, 2003). In the ovary, AMH is exclusively produced by the granulosa cells and inhibits the recruitment of primordial follicles, presumably by preserving the follicular reserve (Bezard et al., 1987; Durlinger et al., 2002, 1999; Gigli et al., 2005; Vigier et al., 1984). Moreover, its expression (beginning in the perinatal period) remains low throughout the reproductive life of females (Munsterberg and Lovell-Badge, 1991; Vigier et al., 1984). In humans, AMH production has been detected as early as 36 weeks of fetal life, albeit at very low levels (Rajpert-De Meyts et al., 1999). Furthermore, experimental evidence has shown that supraphysiological AMH levels in the fetal ovary, comparable to those in male fetuses, lead to germ cell loss and inhibition of aromatase and LH transcription, negatively affecting ovarian development (Josso et al., 1998).

In the antral follicle, only granulosa cells near the oocyte produce AMH, with no expression observed in the atretic follicles or theca cells. In sheep, AMH is detected in granulosa cells both before and after birth; its presence is influenced by the degree of follicular development and age of the animal (Durlinger et al., 1999). Precocious depletion of AMH has been observed in human females; however, the mechanism by which its production is inhibited in granulosa cells by the hormones FSH and estradiol, as well as the stimulus mechanism of its production, remains unknown. However, in humans, BMPs -2, -6, -7, and -15 induce AMH expression in granulosa cells (Ogura-Nose et al., 2012).

In pregnant buffalo (*Bubalus bubalis*) cows, fetal AMH concentrations are expected to increase with gestational age in male fetuses, but remain relatively stable in females. In fetal buffaloes, particularly males, AMH is expressed in the developing gonads, and its expression increases as gestation progresses. Moreover, maternal AMH levels are not anticipated to show a significant correlation with fetal AMH levels.

Currently, there are no studies on the concentration of AMH in adult buffalo cows and their male or female conceptuses, or on the relationship between AMH concentration and the population of Sertoli cells and gonocytes in the testes of male fetuses and primordial follicles in the ovaries of female fetuses, in addition to the immunolocalization of AMH in fetal gonads. The aim of this study was to fill these gaps in our understanding of AMH in fetal development.

## Methods

### Samples

All biological materials utilized in this study were provided to the in vitro fertilization laboratory of the Federal University of Pará by the local SOCIPE slaughterhouse (Frigorífico SOCIPE, Belém, Pará, Brazil). The materials, including blood and fetal gonads were collected from animals that had already been slaughtered, thus obviating the need for approval by the university's ethics and research committee. Samples were collected from 3 to 8-month pregnant buffaloes at the slaughterhouse. The stage of pregnancy was determined by assessing the fetal crown-rump length (CRL), which was considered adequate within the range of 15 to 98 cm (Abdel-Raouf et al., 1974). Blood samples were drawn from cows during exsanguination and from fetuses via cardiac puncture, following slaughter. Gonads were also dissected from the same fetuses.

### Blood collection and AMH assay

Blood was collected into EDTA-coated tubes (26 from pregnant cows, 12 from male fetuses and 14 from female fetuses). Subsequently, the samples were centrifuged at 180g for 5 minutes, and plasma was stored at -20 °C until analysis. AMH levels were determined using the Bovine AMH ELISA kit AL-114 (AnshLabs, city, TX, USA), following the manufacturer’s protocol. Prior to analysis, male samples were diluted 1:100 using the provided diluent. The assay has an analytical range of the assay is 37.5-2400 pg/mL and an analytical sensitivity of 11 pg/mL. The intra-assay coefficients of variation were 2.92%, 2.54% and 3.65% for quality control plasma samples containing 611 and 1259 pg/mL AMH, respectively.

### Fetal gonad processing and morphometry

One gonad from each fetus (12 males and 14 females) was fixed in 10% formaldehyde for 24 hours and then dehydrated in increasing concentrations of alcohol, diaphanized in xylol, infiltrated and embedded in paraffin. Seven-µm thick histological sections of the testes were either stained with hematoxylin and eosin (HE) for morphometry and cell quantification. The morphometry of seminiferous tubules involved measuring the tubular diameter and quantifying Sertoli cells and gonocytes in 20 round transverse sections per animal.

For the ovaries, serial 7-µm thick sections were cut throughout the gonad, and the total number of sections per ovary was counted. Three random sections of each ovary were stained with HE for histological processing, ovarian structure and morphometry. The populations of primordial and primary follicle were quantified according to Gougeon and Chainy (1987).

All analyses were conducted using the Eclipse Ci-E photomicroscope (Nikon Corporation, Tokyo, Japan) coupled with a digital camera (NIKON DS-Ri1, Nikon Corporation, Tokyo, Japan) using NIS-Elements Basic Research software NIKON Version 4.0 (Nikon Corporation).

### Immunohistochemistry

For the immunolocalization of AMH, histological sections of the testes and ovaries were deparaffinized in xylol, rehydrated in ethanol, washed in phosphate buffered saline (PBS), and then incubated in 3% hydrogen peroxide in methanol for 30 minutes. The slides were subsequently immersed in sodium citrate buffer heated to 70 °C for 25 minutes and blocked with 10% normal goat serum (16210072, Invitrogen, Burlington, ON, Canada) for 1 hour. Following this, the slides were incubated overnight with anti-AMH antibody (1:50; SC 28912 - Santa Cruz Biotechnology) according to the manufacturer's instructions. The slides were then washed in PBS containing 0.05% tween (TWEEN 20, 25564, EMS, Hatfield, PA) and post-incubated with peroxidase conjugated anti-rabbit IgG secondary antibody (1:500; sc-2030) for 2 hours. Subsequently, the slides were immersed in DAB (3,3 'diaminobenzidine) solution (750118, Invitrogen, Burlington, ONT, Canada) for 5 minutes, washed in distilled water, dehydrated and covered with a coverslip and entellan. For negative control slides, PBS was used instead of the primary antibody.

Slides were examined using a photomicroscope (NIKON Eclipse Ci-E, Nikon Corporation, Tokyo, Japan) coupled with a digital camera (NIKON DS-Ri1, Nikon Corporation, Tokyo, Japan). We evaluated AMH immunolocalization within the gonads and identified the cell types immunostained at different fetal ages.

### Statistical analysis

Statistical analysis of AMH concentration was preceded by a normality test and conducted using ANOVA with a significance level of 0.05 and Tukey's post-hoc test. For studies on the correlation between AMH and follicular population, simple linear regression analysis was employed to derive regression functions. All analyses were performed using BioEstat5.0 software (Ayres et al., 2007).

## Results

**Figure 1:**
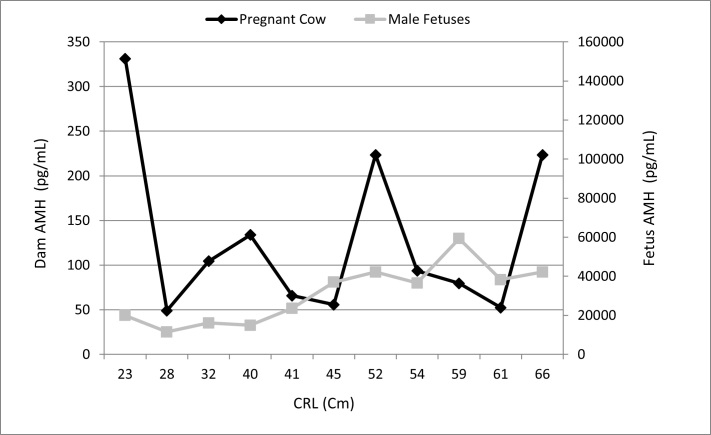
Average anti-mullerian hormone (AMH) concentrations in pregnant buffaloes and their respective male fetuses. Gestational age estimated by the crown-rump length (CRL).

The concentration of AMH in buffalo cows pregnant with male fetuses exhibited significant individual variation, ranging from 330 to 122.66pg/mL at 3 and 8 months of gestational age, respectively. Furthermore, no correlation was observed between maternal and fetal AMH concentrations and gestational age (r = -0.04; p = 0.089). However, AMH concentrations varied significantly (p < 0.05) among male fetuses of different ages, with concentrations increasing with gestational age (Figure 1).

There was significant individual variation in AMH levels among male fetuses of all ages, with concentrations ranging from 158,370 to 976,550 pg/mL. The AMH concentration was significantly higher than the corresponding values in the pregnant cows (Table 1).

**Table 1:** Average Anti-Mullerian Hormone (AMH) plasma concentrations (pg/mL±SD) in pregnant buffalo cows and their male fetuses by gestational age and AMH immunolocalization in the fetal testis.

CRL (*)	**n**	**Mother AMH**	**Fetal AMH**	**Testis AMH immunolocalization**
15-34 (3-4)	3	161.42 (±149.38)	158,370 (±42.10)^a^	NA
35-47 (5)	3	85.03 (±42.56)	251,270 (±111.08)^ab^	+
48-62 (6)	4	112.16 (±76.08)	440,380 (±104.09)^b^	+
63-98 (7-8)	2	122.66 (±0)	976,550 (±45.26)^c^	+

* Fetal age in months. CRL: cross ramp lenght (cm); n: number of fetus analysed; NA: not analyzed; +: Positive; SD: standard deviation. Different superscripts within column denote statistically significant differences (p<0.05).

Our findings suggest a weak positive correlation between plasma AMH concentration in male fetuses and Sertoli cell numbers (r = 0.42, p = 0.34), as well as between Sertoli cell and gonocyte numbers (r = 0.28, p = 0.53). However, owing to the sample size analyzed in our study, the p-value exceeded 0.05; thus, the results failed to confirm our hypothesis. Similar results were observed regarding the correlation between AMH concentrations and gonocyte numbers, where the negative correlation was not substantiated (r = -0.31; p = 0.48), likely due to sample size limitations.

**Figure 2:**
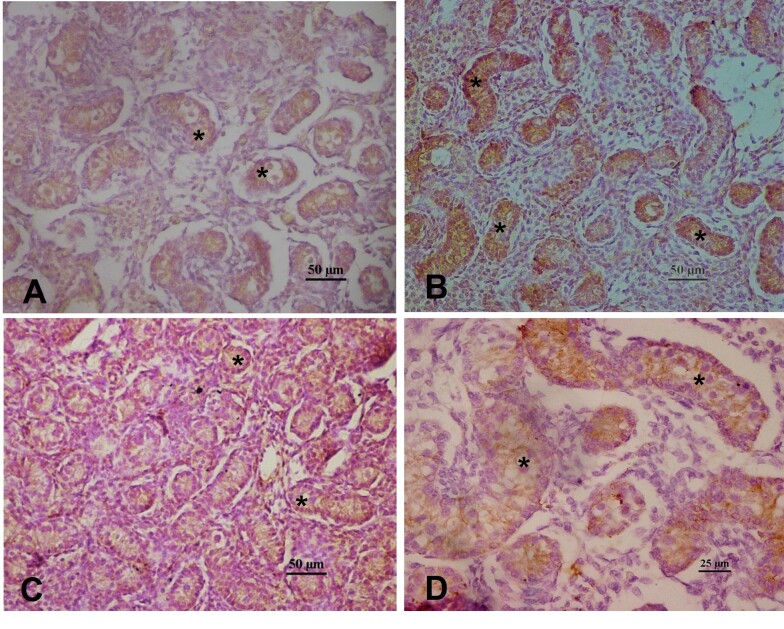
Immunolocalization of AMH fetal buffalo testes at different ages. *Intense immunostaining was observed within the seminiferous tubules restricted to Sertoli cells. (A) 5 months; (B) 6 months; (C) 7 months; (D) 8 months.

Immunohistochemistry analysis of AMH in the fetal testes revealed intense labeling in the cytoplasm of pre-Sertoli cells within the sex cords (developing seminiferous tubules) at all ages. Immunostaining was not observed within the gonocytes or the interstitial tissue (Figure 2).

**Figure 3:**
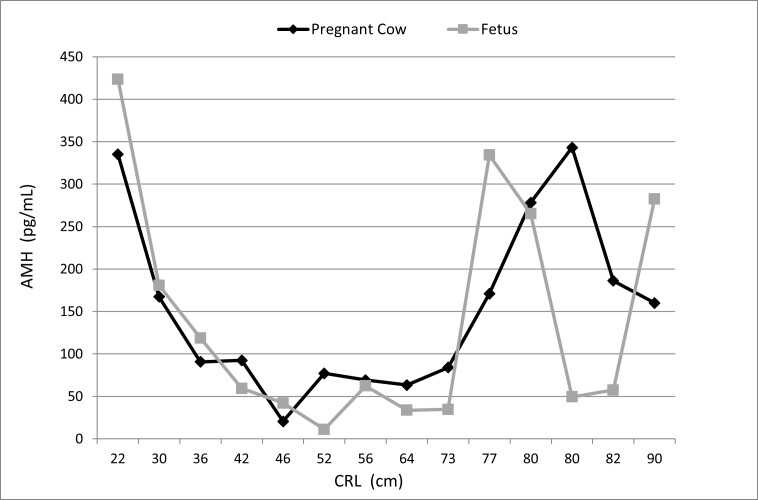
Average anti-mullerian hormone (AMH) concentrations in pregnant buffaloes and their respective female fetuses. Gestational age estimated by the crown-rump length (CRL).

The average AMH plasma concentrations were not significantly different (p<0.05) between pregnant cows and female fetuses across different gestational ages, although significant individual variation was observed, with values ranging from 20 to 408 pg/mL. Similarly, AMH concentrations did not differ (p<0.05) between female fetuses of different gestational ages. This variability may be partly attributed to the considerable differences observed among animals of the same age, with concentrations ranging from 11 to 423 pg/mL (Figure 3).

Data on mothers and female fetuses are summarized in Table 2 and Figure 3.

**Table 2:** Average Anti-Mullerian Hormone (AMH) plasma concentrations (pg/mL±SD) in pregnant buffalo cows and their female fetuses by gestational age and AMH immunolocalization in the fetal ovaries.

**CRL**	**n**	**Mother AMH**	**Fetal AMH**	**IHC AMH ovary fetus**	**Primordial follicles**	**Primary follicles**	**Secondary follicles**	**Antral follicles**
15-34	2	251.2 (±118.7)	302.12 (±171.6)	NA	NA	NA	NA	NA
35-47	3	67.89 (±41.1)	73.45 (±40.3)	+	28.020 (±26.8)	865,78 (±240,6)	NO	NO
48-62	2	73.25 (± 5.5)	36.74 (± 36.3)	+	32.479 (±13.8)	7260,33 (±6.490,)	O	NO
63-69	3	106.09 (±57.1)	134.23 (±173.2)	+	11.581 (±10.811)	800,55 (±698,)	O	O
80-98	4	241.85 (±84.3)	163.59 (±127.5)	+	9.996 (±4.331)	2.348,6 (±659,3)	O	O

CRL: Crown-rump length; n: number of samples; NA: not analyzed; NO: not observed; O: observed; IHC: immunohistochemistry; +: Positive immunostaining. Average ± standard deviation. p>0.05.

Although a positive correlation was noted between maternal and female fetus AMH concentrations (r = 0.58, p = 0.02), there was no correlation between the number of primordial follicles in fetal ovaries and fetal AMH concentrations (r = -0.18; p = 0.57). Owing to challenges in the histological processing of ovaries from 3 to 4 months of age, it was not feasible to conduct histological analysis at this fetal age.

The population of primordial follicles in fetuses at 5 months of gestation also exhibited considerable individual variation, but no statistically significant difference between fetal ages or correlation was observed between AMH concentrations and the population of primordial follicles.

Primary, secondary, and antral follicles were observed in fetal ovaries starting at the fifth, sixth and seventh month of gestation. However, no correlation was found between fetal AMH concentration and the presence of these follicular stages.

**Figure 4:**
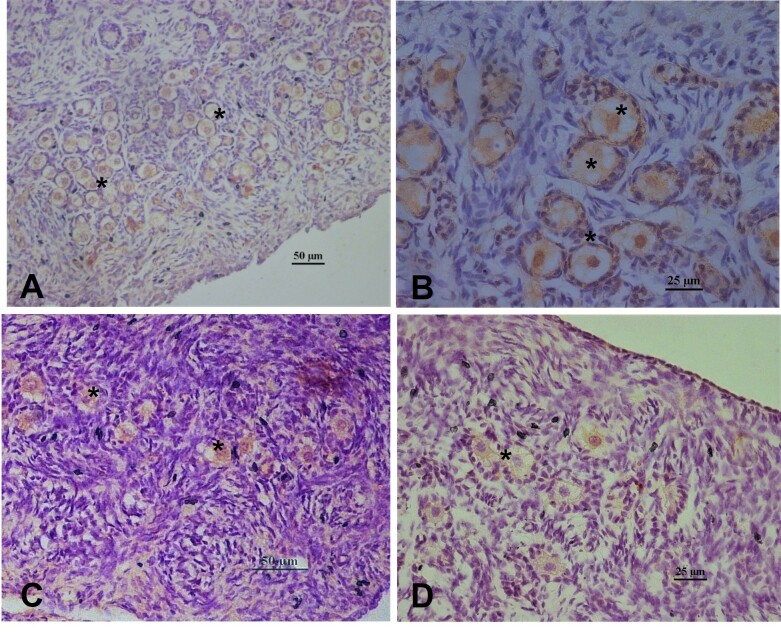
Immunolocalization of AMH fetal buffalo ovaries at different ages. *Intense immunostaining was observed within primordial follicle oocytes. (A) 5 months; (B) 6 months; (C) 7 months; (D) 8 months.

Immunolocalization of AMH in fetal ovaries was observed at all ages evaluated from the fifth month onwards and displayed intense immunostaining within the cytoplasm of primordial follicle oocytes at 5, 6, 7, and 8 months (35–98cm CRL). Marking was mild in the granulosa cells, and no staining was observed in the theca cells (Figure 4).

When comparing AMH plasma concentrations between male and female fetuses, the average plasma concentrations were found to be 15.8 ±4.20 vs 0.302 ± 0.17, 25.06 ± 11.13 vs 0.073 ±0.04, 44.00 ±10.4 vs 0.036 ±0.03, and 97.6 ± 4.52 vs 0.1511 ± 0.13 ng/mL measured at 3-4, 5, 6, and 7-8 months, respectively. This indicates that in the male fetuses the level of AMH was 52, 34, 1,222, and 650 times greater than the corresponding levels in the female fetus at 3-4, 5, 6, and 7-8 months, respectively.

**Figure 5:**
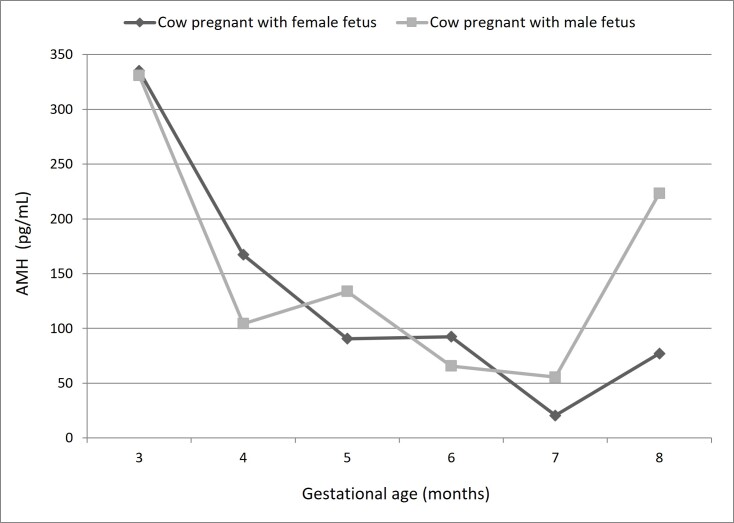
Comparison of plasma AMH concentration in pregnant cow with male or female fetuses of the same approximate gestational age (estimated by the crown-rump length).

When comparing the plasma concentrations of AMH between mothers of male or female fetuses, no significant differences (p<0.05) were observed, suggesting that fetal sex and AMH concentration did not affect circulating AMH concentrations in the mother (Figure 5).

## Discussion

This is the first report on the plasma concentrations of AMH in pregnant buffaloes and their respective conceptuses, including the immunolocalization of AMH in the fetal gonads.

### Plasma AMH in pregnant buffaloes

Previous reports on plasma and/or follicular fluid AMH concentrations in cycling cattle have demonstrated its potential as a reproductive/fertility marker (Batista et al., 2014; Stojsin-Carter et al., 2016; Hirayama et al., 2017). It is also important to consider that genetic differences have been found among different cattle species in AMH measurements (Stojsin-Carter et al., 2016).

AMH concentrations in pregnant buffaloes have not been reported in the literature. However, these results differ from those observed in humans (Pankhurst et al., 2016). Conversely, in humans, AMH concentrations in the maternal serum progressively decrease as pregnancy progresses (McCredie et al., 2017). However, when human recombinant AMH was injected intravenously into mice, it rapidly dispersed into the extracellular fluid space, suggesting that extracellular fluids should be considered when assessing circulating AMH levels (Pankhurst et al., 2014); however, the authors observed that the proportion of pro-AMH (inactive precursor)/total AMH showed little or no variation during gestation. This finding suggests that—due to differences in size between mother and fetus—fetal blood AMH does not appear in the mother's blood; in the case of buffaloes, the maternal extracellular fluid volume is approximately 15 L, whereas in the fetus, the blood volume is 30 mL at the 21st week and increases to 190 mL at the 35th week.

Not only did AMH concentrations show an increasing trend with gestational age in male fetuses, male fetal AMH concentration was 169-813 times greater than that in maternal circulation. Moreover, there were no differences in AMH concentration between pregnant cows carrying male or female fetuses. The lack of correlation between maternal and fetal AMH concentrations is consistent with the inability of this hormone to cross the placenta in cattle, and supports the notion that neither the stage of pregnancy nor the sex of the fetus influences dam AMH concentrations.

The plasma AMH concentrations in pregnant buffaloes were found to differ from the concentrations of AMH in humans with female fetuses, with average AMH concentration of 1.69, 0.80, and 0.5 ng/mL in the 1st, 2nd and 3rd trimester, respectively. Women between 28-34 years of age present a higher concentration of AMH in the first trimester of pregnancy: AMH decreases significantly with advancing gestational age and AMH cannot be used as a parameter of gestational age and ovarian reserve in some circumstances, such as gestation (Köninger et al., 2013).

### AMH in male fetuses

A gradual increase in plasma AMH concentration was observed in male buffalo fetuses with advanced gestational age. This phenomenon aligns with testis development and the proliferation of Sertoli cells, with AMH concentrations at eight months being comparable to those reported in newborn Holstein Friesian bulls (Rota et al., 2002). In contrast, among female fetuses, there was no corresponding increase in AMH concentration with gestational age, although levels were often higher than anticipated when compared with results in other species (Cate et al., 1986).

As an example, during the first trimester of human pregnancies, low AMH levels were only detected in the serum of male fetuses (Cate et al., 1986). In human female fetuses, AMH is not detectable until approximately the 36th week of gestation. However, regardless of species, AMH levels in female fetuses are 35 times lower than those in male fetuses of the same gestational age, as observed in our study. Thus, there may be species-specific aspects governing AMH levels.

To corroborate the origin of AMH in the fetus, we performed immunohistochemical analysis of the fetal gonads. A positive signal was observed in both the developing testes and ovaries, beginning as early as approximately 5 months of gestational age. The presence of AMH in fetal testes from 5 months onwards agrees with that observed in equine fetal testes, where AMH immunoreactivity was detected at 5.5, 10, and 11 months of pregnancy, as well as up to 1 year of age (Ball et al., 2008). However, once the androgen receptors reach an appropriate threshold in Sertoli cells, AMH is inhibited, testosterone reaches the seminiferous tubules, and spermatogonia enter meiosis (Al-Attar et al., 1997). In addition, AMH immunolocalizes to developing Sertoli cells without immunostaining of presumptive gonocytes (Ball et al., 2008).

Conversely, AMH immunolocalization in fetal ovaries showed strong immunostaining in the cytoplasm of oocytes from primordial, primary, and secondary follicles and weak or non-existent labeling in granulosa cells. In humans, AMH has been detected in all oocytes, ovarian stroma, and theca cells along the granulosa layer of growing follicles (Stubbs et al., 2005). In both prepubertal and adult sows, AMH immunostaining was observed in granulosa cells of primordial, primary, preantral, and preovulatory follicles, as well as within oocytes (Almeida et al., 2018). These differences in AMH expression patterns among species have led to speculations on the role of AMH in ovarian physiology, particularly during fetal life (Modi et al., 2006).

Notably, at the mRNA level, Modi et al. (2006) observed that AMH is expressed in the granulosa cells of fetal and neonatal ovaries in primates. These findings suggested that AMH plays a role in regulating folliculogenesis. The authors also mentioned that in human fetal ovaries, AMH expression occurs in the granulosa cells by the 13^th^ week and that this expression continues during follicular formation, where the granulosa cells involve the oocyte to form primordial follicles, concluding that AMH may play a role during ovarian fetal development, which requires further investigation.

In the adult human, the consensus is that AMH plays a role in folliculogenesis and inhibits the recruitment of primordial follicles. The addition of AMH to human ovarian tissue culture medium promotes the recruitment and survival of primordial and early-onset follicles (Schmidt et al., 2005).

### AMH in female fetus

AMH inhibits the growth of primordial follicles (as demonstrated by immunohistochemistry) and is located in the ovarian stromal cells that surround the nests of germ cells and primordial neo-formed follicles. This suggests that, in bovines, stromal cells are the source of AMH, which appears to be different from what is observed in adult animal ovaries, where AMH inhibits the transition from primordial to primary and is produced by granulosa cells from secondary follicles and from the onset of antrum to antral formation. Although the AMH localization sites are different, its role in neonatal and adult ovaries in rat is to inhibit the progression of primordial follicles (Nilsson et al., 2011).

## Conclusions

In conclusion, in pregnant buffaloes, maternal plasma AMH concentrations showed no correlation with AMH concentrations in their respective fetuses, suggesting that AMH does not cross the placental barrier. AMH concentrations were significantly higher in male fetuses, showing an increase according to fetal age, than in female fetuses, in which no correlation was observed between AMH concentration, follicular population, or the presence of antral follicles. However, further studies are required to better understand the role of AMH in the development and physiology of the gonads, gametogenesis, and reproduction in buffaloes.
